# 

**Published:** 1880-01

**Authors:** 


### CONTENTS.

COMMUNICATIONS.

Salivary Calculus and its Concomitants .......... 7
What was the cause of the Death of Geo. A. Gardner? .......... 15
Discussions at the Ohio State Dental Society .......... 21

SCIENTIFIC NEWS.

Edison's Electric Lamp .......... 36
Miscellaneous Notes .......... 41
Dr. S. S. White's Death .......... 43

EDITORIAL.

Bibliographical .......... 47

